# Traditional healers and the potential for collaboration with the national tuberculosis programme in Vanuatu: results from a mixed methods study

**DOI:** 10.1186/1471-2458-14-393

**Published:** 2014-04-23

**Authors:** Kerri Viney, Penelope Johnson, Markleen Tagaro, Saen Fanai, Nguyen N Linh, Paul Kelly, David Harley, Adrian Sleigh

**Affiliations:** 1 Secretariat of the Pacific Community, Public Health Division, BP D5, Noumea Cedex 98848, New Caledonia; 2 National Centre for Epidemiology and Population Health, Australian National University, Building 62, Corner of Eggleston and Mills Roads, Canberra ACT 0200, Australia; 3 School of Culture, History and Language (RSPAS), College of Asia and the Pacific, Australian National University, Canberra ACT 0200, Australia; 4 Ministry of Health, PB 9009, Port Vila, Vanuatu; 5 Global TB Programme, World Health Organization; formerly from The Division of Pacific Technical Support, World Health Organization Representative Office in the South Pacific, Suva, Fiji; 6 Population Health Division, ACT Health, ACT Government, GPO Box 825, Canberra City ACT 2601, Australia

**Keywords:** Tuberculosis, Pacific, Traditional healers, Health care

## Abstract

**Background:**

This study was conducted in the Pacific island nation of Vanuatu. Our objective was to assess knowledge, attitudes and practice of traditional healers who treat lung diseases and tuberculosis (TB), including their willingness to collaborate with the national TB programme.

**Methods:**

This was a descriptive study using both qualitative and quantitative methods. Quantitative analysis was based on the responses provided to closed-ended questions, and we used descriptive analysis (frequencies) to describe the knowledge, attitudes and practice of the traditional healers towards TB. Qualitative analysis was based on open-ended questions permitting fuller explanations. We used thematic analysis and developed *a posteriori* inductive categories to draw original and unbiased conclusions.

**Results:**

Nineteen traditional healers were interviewed; 18 were male. Fifteen of the healers reported treating *short wind* (a local term to describe lung, chest or breathing illnesses) which they attributed to food, alcohol, smoking or pollution from contact with menstrual blood, and a range of other physical and spiritual causes. Ten said that they would treat TB with leaf medicine. Four traditional healers said that they would not treat TB. Twelve of the healers had referred someone to a hospital for a strong wet-cough and just over half of the healers (9) reported a previous collaboration with the Government health care system. Eighteen of the traditional healers would be willing to collaborate with the national TB programme, with or without compensation.

**Conclusions:**

Traditional healers in Vanuatu treat lung diseases including TB. Many have previously collaborated with the Government funded health care system, and almost all of them indicated a willingness to collaborate with the national TB programme. The engagement of traditional healers in TB management should be considered, using an evidence based and culturally sensitive approach.

## Background

Tuberculosis (TB) is a significant public health problem in many Pacific island countries, including in Vanuatu [1]. TB incidence rates vary greatly between Pacific island nations [2], however all Pacific Island countries have a dedicated national TB programme (NTP) for TB prevention, treatment and management.

Historically, NTPs have attempted to determine the contribution of traditional healers to TB management, with the aim to involve them in TB care [3-5]. Many NTPs have also investigated traditional healers’ and patients’ knowledge, attitudes and practices regarding TB. Most of this research is from Africa and South Asia [3,4,6-9]. These studies have demonstrated: 1) a delay in TB diagnosis due to consultation with a traditional healer, with consequent prolongation of the period until radiological and microbiological confirmation [10-12], and 2) the occasional success of NTPs to engage traditional healers in TB care [5,8,9]. This research has focused on traditional healers in Africa, India and Nepal [3,8,13,14], but there is little Pacific research, and none that focuses on co-operation between Western health care systems and traditional healers. This relationship is not always straightforward due to potential misunderstandings on both sides. For example, health authorities may object to collaboration with traditional healers, as their roles are often conflated with negative images of “black magic”, and sorcery, which are opposed to modern notions of benevolent biomedicine.

In the Republic of Vanuatu medicine, religion, magic, and modernity are intertwined [15]. Treatment choices involve the traditional and the modern, and individuals pragmatically decide who to consult, with access and affordability prominent determinants of choice [16,17]. Therefore, initial selection of health care provider may have little to do with the perceived healing capabilities of traditional healers or the acceptance or rejection of Western medicine, but may reflect the cost of accessing health care, or proximity of health services [16,17]. A traditional healer is usually first consulted, as they are typically local, familiar, affordable, dispense medication for symptoms, and provide locally comprehensible and culturally appropriate explanations of disease causation [4,7,8,17].

Standard short course treatment with a combination of first line anti-TB drugs is the internationally recognised standard of care for patients diagnosed with TB [18]. Traditional healers treat lung infections with local medicine (including leaves) [19], but little is known about the contribution of traditional healers towards TB management in the Pacific, or in Vanuatu. The NTP in Vanuatu is also interested in assessing the potential role of traditional healers in community based management of TB. We therefore decided to assess the knowledge, attitudes and practice of traditional healers towards TB in Vanuatu. Specific objectives were to describe 1) the characteristics of traditional healers from four sites, 2) their treatment of lung diseases, 3) their treatment of TB, and to assess: 4) the willingness of healers to collaborate with the NTP, and any incentives required for doing so.

## Methods

### Study design

This study used a mixed methods study design (qualitative and quantitative methods). We used ethnographic methods, incorporating indigenous researchers to conduct individual interviews that documented the knowledge, attitudes and behaviours of traditional healers towards TB in Vanuatu. This approach was used as a way of presenting a local understanding of lung health and TB. Semi-structured questionnaires contained both closed and open-ended questions which were used to determine knowledge, attitudes and practices regarding the cause and treatment of lung diseases, including TB. Using a comparative method, issues were examined diachronically; comparing what people used to do and what they do now, and synchronically across district settings.

Our analysis used a grounded-theory approach, where interview data were organised thematically, creating various unique categories, which in turn were used to explain and create a theoretical perspective. The key themes identified were interwoven throughout the Results section as they related to certain questions or groups of questions, and they were further explored in the context of other study findings as part of our discussion. Our study adhered to the RATS guidelines for qualitative research (i.e. Relevance, Appropriateness, Transparency and Soundness of interpretive approach) [20].

### Setting

**Figure 1:**
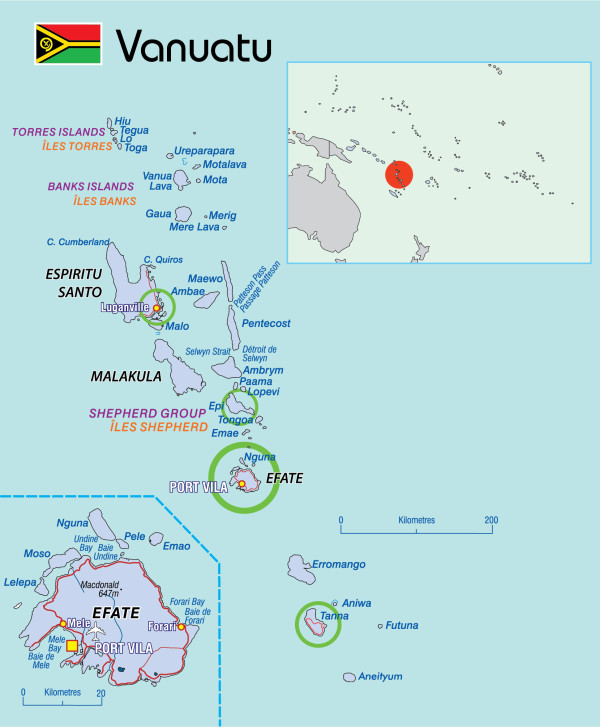
**Map of Vanuatu, showing the four study sites (circled in green).** Source: Secretariat of the Pacific Community*.*

The study was conducted in the Republic of Vanuatu; an island nation in the South Pacific Ocean (Figure 1). It is an archipelago of 82 islands, of which 65 are inhabited, and the country is divided into six provinces (Figure 1). The population of approximately 260,000 people is 98.5% ni-Vanuatu, a distinct ethnic group within the family of Melanesian descent [21].

For the most part, Western health care in Vanuatu is provided by the national Government, with little private sector involvement. Health care delivery occurs in hospitals, health centres, dispensaries and aid-posts throughout the country [22]. In 1993, the Government introduced free health care, however patients may still have to pay a contribution fee for outpatient services [22].

In parallel, traditional medicine is provided by traditional healers (*klevas*) throughout Vanuatu. The World Health Organization (WHO) estimate that there are approximately 200 traditional healers in Vanuatu [21]. This informal system of healthcare is termed *kastom* medicine. *Kastom* is “the word that people in Vanuatu use to characterise their own knowledge and practice in distinction to everything they identify as having come from outside their place” [23].

Vanuatu reports approximately 110 cases of tuberculosis each year and is regarded as a medium burden country in the Pacific context [2]. The WHO estimate that 67-80% of all TB cases in Vanuatu are detected, and once diagnosed and treated, Vanuatu consistently reports treatment success rates of 80% or higher [2].

### Participants

The participants were traditional healers enrolled from one of four study sites in Vanuatu: 1) Port Vila, 2) Luganville, Santo 3) Tanna, and 4) Epi. These sites were chosen because a large proportion of TB patients have come from these areas in recent years (72% over the last five years), and they comprise both urban and rural populations. Participants self-identified as currently practising traditional healers. They were recruited using a snowball sampling method, where a well-known healer at each site was approached initially, and he or she then referred new participants. Interviews continued until all categories and themes were saturated, and no new information emerged. No material incentives were provided. Interviews were conducted between October 2011 and February 2012.

### Data collection

A semi-structured questionnaire was designed for the study. It contained three sections: 1) demographic information, 2) information on lung diseases, and 3) treatment of TB and potential collaboration with the NTP. Questions were closed and open ended, and interviewers were able to ask for additional information where needed. The questionnaires were pilot tested prior to commencement of the study. All participants were interviewed by trained ni-Vanuatu nurse interviewers, and were conducted in the lingua-franca Bislama, or in the local language. All interviews were recorded and were later transcribed and translated into English.

### Analysis and statistics

#### Quantitative analysis

Quantitative data were double entered into EpiData version 3.1. Descriptive analyses were carried out in EpiData Analysis version 2.2.2.178. Quantitative analysis was based on the responses provided to closed-ended questions, which had a number of pre-defined answers.

### Qualitative analysis

We adopted a mixed interdisciplinary and non-representative approach, which included the description of physical, biological and social phenomena. We used thematic analysis and developed *a posteriori* inductive categories to draw original and unbiased conclusions. Qualitative analysis was based on open-ended questions, permitting fuller explanations.

### Ethical considerations

Ethics approval was provided by the WHO-Western Pacific Region Office Ethics Review Committee and the Australian National University Human Research Ethics Committee. The Vanuatu Ministry of Health and the Vanuatu National Cultural Council also provided approval. The traditional healers provided written consent after receiving written and oral information about the study.

## Results

### Characteristics of the healers

Nineteen healers were interviewed; 18 were male (Table 1). Seven traditional healers were interviewed from Tanna, four from Luganville, Santo, five from Port Vila and three from Epi (Table 1). Just under half (7) of the traditional healers preferred to be known by the name “*kastom* doctor/*dokta*” and two thirds (10) had been a traditional healer for ten years or more (Table 1). Most of the healers reported living in the same community long-term (i.e. ten years or more). The communities they served ranged from 50-3,000 people (Table 1). Of those who could report how many patients they treated, almost three quarters (9) said they treated more than 100, with a range of 50-4000 patients per year (Table 1).

**Table 1:** Demographic and other characteristics of 19 traditional healers in Vanuatu

**Characteristic**^**§**^	**Number**	**Percentage**
Sex (n = 19)		
Male	18	95
Age group (n = 19)		
18-29 years	1	5
30-39 years	1	5
40-49 years	3	16
50-59 years	3	16
60 years and over	4	21
Age group not recorded	7	37
Location (n = 19)		
Epi	3	16
Port Vila	5	16
Santo	4	21
Tanna	7	37
Name of healer (n = 17)		
Kleva*	1	6
*Kastom^ dokta*^*#*^ (or *dokta*)	7	41
Healer	1	6
Other	8	47
Length of time as healer (n = 15)		
1-4 years	2	13
5-9 years	3	20
10-19 years	4	27
20 years of more	6	40
How learned to be a healer (n = 19)		
Ancestors	1	5
Grandparents	3	16
Parents	4	21
Spirit or God	5	26
Other	6	32
Number of people in community (n = 15)		
51-100	4	27
101-200	2	13
200 or more	9	60
Number of people treated in a year (n = 12)		
Less than 50	1	8
51-100	2	17
101-200	2	17
201 or more	7	58

^**§**^n = number of valid responses. All percentages were calculated as a proportion of the number of valid responses, which are indicated in brackets.

*Kleva: Bislama word for a traditional healer or traditional medical practitioner.

^*Kastom*: The word that people in Vanuatu use to characterise their own knowledge and practice in distinction to everything they identify as having come from outside their place.

^#^*Dokta*: Bislama translation of the word “doctor”. *Kastom dokta* refers to a traditional healer in Vanuatu.

Eight of the healers learned their practice from their ancestors while others learned from a spirit, God or by other means (Table 1). Many of the healers recounted that they first learned how to be a traditional healer when confronted by an illness.

“Mi bin lanem ol samting ia from wan taem we angkel blong mi hem i bin fuldaon mo brekem han blong hem mo mi garem hem mo go luk wan kleva. Mi askem kleva ia blong i tijim mi mo mi bin pem hem wetem wan buluk mo wan stamba blong kava”.

*“I have learned mine through my uncle who fell and broke his arm and I took him to my custom doctor. That was where I asked the man to teach me and I bought it [the knowledge] with a cow and a kava stem”* (Male traditional healer, TH4).

Many of the healers described that they could treat “lots of illnesses” or “everything” while others were more specific and described a range of illnesses and symptoms that they routinely treated.

“Suga, main wan ia nao, hae blad, cancer, kidney problem, heart problem, waet liver problem, hemia nao olgeta main wan we ol man I stap luk mi from”.

*“Most people come to see me for diabetes. Also high blood pressure, cancer, kidney problems, heart problems, white-liver problems, these are the main ones”* (Male traditional healer, TH9).

Illnesses and symptoms that were commonly reported were: hypertension, cancer, diabetes, headaches and “*kastom* illnesses”. We did not know if the biomedical terms were part of the healer’s diagnostic vocabulary, or if they adopted these labels from their patients.

Four of the healers said that they would not treat TB; four were also reluctant to treat severe blood loss. While some healers stated that they would treat “everything”, others said that they avoided certain conditions such as: yaws, “chronic conditions”, AIDS, diabetes, paralysis, “heart problems”, “wet dreams” and asthma.

### Treatment of lung diseases

When asked if they treated *short wind*, 15 (83%) responded that they did (Table 2). *Short wind* was frequently interpreted as asthma by the healers, although not always. *Short wind* was attributed to food (5, 31%), alcohol or smoking (3, 19%), and pollution by menstrual blood (3, 19%) (Table 2):

**Table 2:** Reported practice of treatment of lung diseases by traditional healers in Vanuatu

**Description of practice**^**§**^	**Number**	**Percentage**
Treat *short wind* (n = 18)	15	83
How do people get this sickness^#^ (n = 16)		
Alcohol/ smoking	3	19
Food	5	31
Hereditary	1	6
Other natural cause	2	13
Other spiritual cause	2	13
Sexual intercourse with menstruating women	3	19
Symptoms of short wind*		
Cough	8	42
Discoloured eyes	3	16
Fever	2	11
Haemoptysis	3	16
Fatigue	5	26
Shortness of breath	8	42
Weight loss	5	26
Other symptoms	10	52

^**§**^n = number of valid responses. All percentages were calculated as a proportion of the number of valid responses, which are indicated in brackets.

^#^The healers were asked to give the single most important response for the question: How do people get this sickness?

*The healers described more than one symptom for *short wind*, therefore these percentages do not add up to 100.

“Mi save se man we i kasem sotwin hem i mas stap silip blong gat seks wetem waef o wan woman we i stap luk sikmun from toti blad i go insait lo bodi blong hem mekem se hem i sik”.

*“I know that a person who gets sick with asthma has been having sex with his wife or a woman who has been menstruating, that is the infected blood that gets into his body making him sick of shortness of breath”* (Male traditional healer, TH4).

Some healers mentioned that *short wind* was like TB, or led to TB.

### Treatment of TB and collaboration with the national TB programme

Three of the healers stated that they had treated TB, another said he would collaborate with the hospital on TB and another stated that he had treated “unclean lungs”. Two healers stated that they treated cough. Just over half of the healers (10, 53%), said that, if needed, they would treat TB with leaf or *kastom* medicine, while others said that they would treat it with massage, water and prayer (Table 3). Leaves were rubbed or placed on a patient, administered in a drink or given to patients to ingest or chew. The healers stated that there were different kinds of leaves for TB if one symptom was predominating, i.e. a leaf for cough, another for haemoptysis etc., and that various combinations of leaves could be offered together. Further, if a patient was not improving the leaf could be changed:

**Table 3:** Reported practice of treatment of tuberculosis by traditional healers in Vanuatu

**Description of practice**^**§**^	**Number**	**Percentage**
Treatment of TB^#^ (n = 19)		
*Kastom* medicine*	4	21
Leaf medicine^	6	32
Massage	2	11
Water	1	5
Don’t treat	2	11
Send to hospital	2	11
Other treatment	2	11
Treatment of strong wet cough^#^ (n = 19)		
*Kastom* medicine	4	21
Leaf medicine	6	32
Massage	1	5
Water	2	11
Don’t treat	4	21
Send to hospital	1	5
Other treatment	1	5
Referral practices (n = 16)		
Refer patients for wet cough	12	75
Refer patients to health clinic	11	69
Work practices		
Work with health clinic (n = 15)	9	60
Willingness to work with NTP (n = 18)	18	100
Conditions for working with NTP (n = 7)^‡^		
No conditions required	2	29
Money	2	29
Token of appreciation	2	29
House	1	14

^**§**^n = number of valid responses. All percentages were calculated as a proportion of the number of valid responses, which are indicated in brackets.

^#^The healers were asked to provide the single most important response to these questions.

*Kastom medicine: *Kastom* is the word that people in Vanuatu use to characterise their own knowledge and practice in distinction to everything they identify as having come from outside their place. *Kastom* medicine is used to denote traditional medical practice in Vanuatu.

^Leaf medicine: Traditional medicine used by traditional healers (*klevas*) in Vanuatu, comprising various leaves and other ingredients, which are ingested, chewed, rubbed or placed on the body.

^‡^These percentages do not add up to 100 due to rounding and the small numerator and denominator.

“Blong ol kaen man olsem ia, bae mi givim tritmen blong sik fiva fastaem bifo mi lukluk lo wet long bodi blong hem. Blong kaen sik we ol i kof, mi gat wan difren lif mo tritmen blong hem. Sapos wan sik man i stap go bunbun tumas, bae mi mas givim meresin blong i leftemap wet blong hem mo sapos kof blong hem i no go daon, bae mi mas givim tritmen blong bodi wet blong hem fastaem bifo mi lukluk lo kof we hem i gat. Hemia i blong jenisim blad i go klin bakegen, taswe hem i save gat paoa bakegen. No, blo kof wetem blad, hemia i gat narafala meresin blong hem bakegen”.

*“But for these kinds of people I would treat the fever separately and then his body weight. With cough I have a different leaf or treatment for that… If the patient is going skinny I give medicine for losing weight, if the cough is going down then I might have to treat his body weight first before the cough. This [is] to try and change the blood to fresh one so that he can regain his strength. [Also] for coughing up blood there is also a different medicine”* (Male traditional healer, TH4).

While the majority of healers responded that they would treat a wet-cough with leaf medicine (10, 53%), four said that they could not treat a strong wet-cough but would refer these patients to the hospital (Table 3). Three quarters (12, 75%), of the healers had referred someone to a hospital for a strong wet-cough (Table 3). Further, just over half of the healers (9, 60%), reported a previous collaboration with the Government health care system, while six (40%) had never collaborated (Table 3).

One of the main points of interest in this study was whether the healers would be willing to collaborate with the NTP and under what circumstances. All of the healers who responded to this question (n = 18) indicated a willingness to work with the NTP. The remaining healer was not asked the question (Table 3).

Most, however, did not respond when asked about conditions under which they would work with the NTP. Those who responded (n = 7) indicated that a small token of appreciation including money, would be appreciated:

“Suga, main wan ia nao, hae blad, cancer, kidney problem, heart problem, waet liver problem, hemia nao olgeta main wan we ol man I stap luk mi from”.

*“If we were to help each other and you thought that you would want to pay me then that would be up to you and I would like to learn more about TB because this is a very strong sickness”* (Male traditional healer, TH13).

Two healers said that no payment (monetary or otherwise) would be needed.

## Discussion

This study contributes towards a better understanding of the knowledge, attitudes and self-reported behaviours of a group of traditional healers towards TB in Vanuatu. We interviewed nineteen traditional healers who came from different provinces and areas in Vanuatu with a TB incidence higher than the national average, and who therefore may have treated persons with presumptive or active TB. Just over half of the traditional healers (10, 53%), said they would use leaf or *kastom* medicine for someone with presumptive TB. It is likely that some of these treatments are partially effective, as anti-mycobacterial activity has been noted in plants from Vanuatu [24-27].

### Key themes identified

The key themes that emerged from many of our questions included a connection between blood (particularly menstrual blood) and disease causation; compounding or ignoring symptoms with other lung diseases; use of biomedical ailment terminology that may or may not have any relation to the true biomedical condition being described; belief in the healers capacity to cure TB with local (i.e. leaf and clay) treatment; and consequential delays in referral to TB services.

### Traditional healers

Ni-Vanuatu traditional healers are generally well-respected, learned and skilled people who treat illness and whose services are widely used [19]. In a study of inpatients and staff from Vila Central Hospital, 86% reported using *kastom* medicine at least once, with 60% reporting use in the past year [19].

Traditional healers use either acquired or inherited knowledge, or divinely given insight to diagnose and treat social and health problems, and they use prayer, massage and leaf medicine for cure and prophylaxis [28]. In Vanuatu, they often combine “botanical expertise with an ability to diagnose illnesses using supernatural powers” [29]. They classify illnesses into categories (i.e. of seriousness and treatability) and many use some type of biomedical terminology to describe the illness. They understand that the causative agent is a pathogen, a social taboo or transgression, and the solution is often based on the traditional healer’s ethno-botanical and spiritual knowledge. Some may profit financially, materially or socially from consultations, but for many it is a calling and is done for the common good [30]. Some traditional healers may refuse monetary payment because it may appear that their exclusive knowledge has been “bought out”, thus affecting its efficacy (M Garde; Anthropologist, Personal communication).

The *kleva’s* role is as a benevolent healer, with no connotations of sorcery. The *kleva*s are clearly identified public practitioners whose activities are open to public scrutiny, while the identity of sorcerers is unknown or suspected by rumour (T Ludvigson; Anthropologist and Vanuatu expert, Personal communication).

Sorcery (*posen* or *nakaimas*), does exist in Vanuatu, “forming an invisible background to social life” and affecting life on a daily basis [15] but it is not the same thing as traditional medicine. Sorcery has moved from being a power of designated sorcerers into the hands of the general populace and today it is generally used for power, mischief and vengeance [31]. Historically, it represented a legitimate use of power by influential men, working for the public interest. It was only after the introduction of Christianity that sorcery became less of a legitimate method of control and was seen as “evil and immoral” [32]. However both sorcerers and traditional healers use powers beyond the comprehension of lay-persons (T Ludvigson; Anthropologist and Vanuatu expert, Personal communication) and traditionally, Melanesian people have not drawn distinctions between the natural and supernatural.

Despite their good standing in the community, traditional healers may be regarded with suspicion by Western medical practitioners. For example, Western medical practitioners can have a dualistic understanding of traditional healers as both wise, skilled healers (this understanding applies to the majority of healers) and charlatans (applicable to the minority), which can lead them to wonder about the healer’s intentions. However, their practices are well established in Vanuatu, and the use of traditional medicine is common [19].

### Treatment of lung diseases and TB

The majority of the healers in our study treated *short wind*, which was commonly interpreted as asthma (although sometimes as influenza). *Short wind* was attributed to food, alcohol, sexual intercourse with a menstruating woman, or a range of other physical and spiritual causes. This is consistent with other studies, whereby the causation of illness is attributed to natural elements, a spiritual cause, or breaking sexual taboos [4,6,33].

### Collaboration with the national TB programme

Despite their different understanding of disease causation and their different approaches to treatment, the healers in our study indicated a willingness to collaborate with the Government funded NTP.

Traditional healers have not been engaged with TB care in the Pacific, however in other countries there have been concerted efforts towards such engagement. In India, the NTP evaluated the acceptability of traditional healers as directly observed treatment providers and found that 84% of patients preferred a traditional healer to their current care provider [3]. In South Africa, traditional healers were engaged to provide supervision of TB medications, and results of TB treatment supervised by traditional healers were as good as those supervised by government health-care workers and other treatment supervisors [8]. Further, 80% of TB patients indicated high levels of satisfaction with the care provided by the traditional healers [8]. Another bio-medical training model for traditional healers found that the trained traditional healers in rural Nepal had practiced modern treatment using first-aid kits and were more likely to refer patients to Government health-care workers [14]. Trained traditional healers in Nepal also provided culturally acceptable health education on HIV-AIDS, distributed condoms and helped to reduce HIV-AIDS related stigma [13]. In another study conducted in The Gambia, traditional healers were engaged to refer persons with presumptive TB to the NTP [5]. In Bangladesh, informal village doctors supervised treatment for 20-45% of all TB patients over a six-year period and treatment outcomes were good [34]. Despite these reports, a systematic review which assessed interventions for educating traditional healers about sexually transmissible infections and HIV concluded that it was “difficult to be certain about the efficacy of interventions for educating traditional healers in the fundamentals of sexually transmissible infection and HIV medicine” [35].

Our findings provide evidence that collaboration between the Vanuatu Ministry of Health and traditional healers is feasible. We have presented evidence that in other countries such collaborations have been beneficial for TB management. However, the legal framework for traditional medicine may require review; Section 18 of the Vanuatu Health Practitioner’s Act of 1984 (amended in 1985), states that it is an offence for a non-registered person to practice medicine or claim to be registered to practice [36]. Traditional healers do not practice medicine in a Western sense, although they often treat illnesses either diagnosed by Western medical practitioners or by themselves, and incorporate biomedical names into their practice. In many countries traditional healers are registered [6,7], and this could be considered in Vanuatu.

### Limitations

The study used a convenience sample which included a small sample of 19 traditional healers in Vanuatu, and the results may not be generalisable to all traditional healers in Vanuatu. Further, a more comprehensive and representative sampling method may have allowed us to assess the impact of regional variations in knowledge, attitudes and beliefs about TB. There may have been bias introduced because TB nurses employed by the Ministry of Health conducted the interviews, and traditional healers may have provided different information to these interviewers, than to others. Our study explored TB treatment behaviours, and we analysed self-reported behaviour. Some management and diagnostic strategies may, as a consequence, be under or over-reported.

## Conclusions

Traditional healers play an important role in health care in Vanuatu and they treat a range of illnesses and symptoms. They claim to frequently treat lung diseases including asthma and tuberculosis. Many have previously collaborated with the Government funded health care system, and they all indicated a willingness to collaborate with the NTP. The engagement of traditional healers in TB management could improve TB case detection and care in Vanuatu. Further, engagement of traditional healers could reduce delays in the time to TB diagnosis and treatment due to early referral of persons with presumptive TB to the health care system. Engagement of *klevas* with the NTP would also ensure that patients receive appropriate curative TB treatment early, thereby reducing the time that partially effective or ineffective treatment is given. Traditional healers could also be engaged to provide directly observed therapy, which would benefit patients living in remote areas including outer islands of Vanuatu, which may be far from an aid post. In Vanuatu, we assert that utilisation of traditional healers should be considered, using a culturally sensitive and evidence based approach.

## Abbreviations

AIDS: Acquired immune deficiency syndrome; HIV: Human immune-deficiency virus; NTP: National tuberculosis programme; TB: Tuberculosis; TH: Traditional healer; WHO: World Health Organization.

## Competing interests

The authors declare that they have no competing interests.

## Author’s contributions

KV conceived and designed the study, analysed the data and drafted the first version of the paper. PJ designed the study, advised on data analysis and interpretation and drafted the manuscript. NNL assisted with the study design and provided input into data interpretation and revised the manuscript. MT conceived the study, assisted with study design, co-ordinated data collection, assisted with analysis and interpretation of the data and revising the manuscript. SF collected data and assisted with data interpretation and revising the manuscript. AS assisted in the conceptual phase, provided advice on data analysis and interpretation, and revised the manuscript. DH also provided advice on data analysis and interpretation, and revised the manuscript. PK provided advice on data analysis and interpretation and revised the manuscript. All authors read and approved the final manuscript.

## Pre-publication history

The pre-publication history for this paper can be accessed here:

http://www.biomedcentral.com/1471-2458/14/393/prepub
